# Ectopic Odorant Receptor Responding to Flavor Compounds: Versatile Roles in Health and Disease

**DOI:** 10.3390/pharmaceutics13081314

**Published:** 2021-08-23

**Authors:** Tao Tong, Yanan Wang, Seong-Gook Kang, Kunlun Huang

**Affiliations:** 1 Beijing Advanced Innovation Center for Food Nutrition and Human Health, College of Food Science and Nutritional Engineering, China Agricultural University, Beijing 100083, China; wangyanan@cau.edu.cn; 2 Department of Food Engineering, Mokpo National University, Muangun 58554, Korea; sgkang@mokpo.ac.kr; 3 Key Laboratory of Safety Assessment of Genetically Modified Organism (Food Safety), Ministry of Agriculture, Beijing 100083, China; 4 Beijing Laboratory for Food Quality and Safety, Beijing 100083, China

**Keywords:** odorant compounds, ectopic odorant receptor, G protein-coupled receptor, cyclic adenosine monophosphate, ectopic function

## Abstract

Prompted by the ground-breaking discovery of the rodent odorant receptor (OR) gene family within the olfactory epithelium nearly 30 years ago, followed by that of OR genes in cells of the mammalian germ line, and potentiated by the identification of ORs throughout the body, our appreciation for ORs as general chemoreceptors responding to odorant compounds in the regulation of physiological or pathophysiological processes continues to expand. Ectopic ORs are now activated by a diversity of flavor compounds and are involved in diverse physiological phenomena varying from adipogenesis to myogenesis to hepatic lipid accumulation to serotonin secretion. In this review, we outline the key biological functions of the ectopic ORs responding to flavor compounds and the underlying molecular mechanisms. We also discuss research opportunities for utilizing ectopic ORs as therapeutic strategies in the treatment of human disease as well as challenges to be overcome in the future. The recognition of the potent function, signaling pathway, and pharmacology of ectopic ORs in diverse tissues and cell types, coupled with the fact that they belong to G protein-coupled receptors, a highly druggable protein family, unequivocally highlight the potential of ectopic ORs responding to flavor compounds, especially food-derived odorant compounds, as a promising therapeutic strategy for various diseases.

## 1. Introduction

Odorant receptors (ORs), which were originally discovered by Richard Axel and Linda Buck in 1991 and constitute the largest members of G protein-coupled receptors (GPCRs) superfamily, have long been thought to function as chemosensor in the olfactory epithelium (OE) where they discriminate and detect volatile odorants. There are roughly 390 in humans and more than 1000 OR genes in mice [1,2,3]. In olfactory sensory neurons (OSNs) of the OE, cyclic adenosine monophosphate (cAMP)-dependent pathway regulates canonical OR signaling: ORs couple to olfactory G protein alpha subunit (Gα_olf_), leading to the activation of adenylyl cyclase 3 (Adcy3, also known as ACIII) and the generation of cAMP [4]. From the standpoint of food sensory science, the olfactory perception is known to play a pivotal role in food flavor, a critical component affecting food acceptance and consumption, as myriad volatile odorants released from foods are detected by ORs expressed in OSNs.

Although ORs are initially described to be restricted only to the OE where they are specific to classic sensory physiology, a growing number of studies have indicated an ectopic expression of ORs in a variety of nonsensory tissues such as testis [5], gut [6], kidney [7], heart [8], muscle [9], pancreas [10], lung [11], liver [12], cerebral cortex [13], skin [14], and blood leukocytes [15,16,17] based largely on (q)RT-PCR or microarray analyses. These ectopically expressed ORs have traditionally been thought to exist without any functional significance and their potential as therapeutic strategies is considered to be limited because they have not been implicated in the pathology of any common disease. Nevertheless, this view is now changing as an increasing number of ectopic ORs, responding to various ligands including specific food-related flavor compounds and gut microbiota-derived metabolites have been reported to exert an important regulatory role in a diverse range of physiological or pathological processes such as renin secretion, cancer growth, triglyceride metabolism, hepatic lipid accumulation, and mitochondrial biogenesis [7,12,18,19,20]. These observations expand our appreciation of the functions of ORs in the modulation of whole-body homeostasis and highlight the potential of ectopic ORs as therapeutical targets and promising markers in the management of human disease in addition to their involvement in the flavor and fragrance industry.

In this review, we scrutinize the literature that has formed our knowledge and understanding about ectopic ORs with regard to their distinct biological functions in response to flavor compounds including various food-derived odorants and downstream signaling pathway underlying their function. The potential therapeutic applications of the ectopic ORs in a variety of diseases will be discussed. We also give a brief overview of the currently identified odorant ligands for human ORs and future directions in the research of ectopic ORs.

## 2. Main Olfactory System

**Figure 1:**
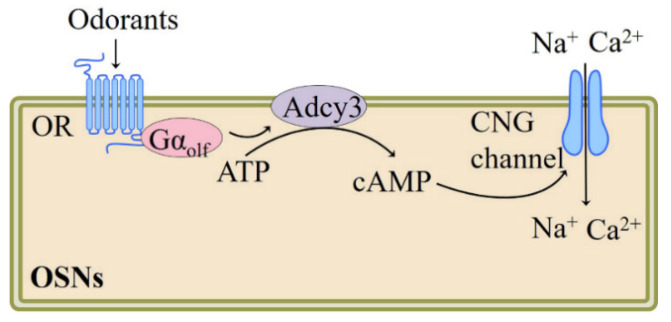
Schematic model of the olfactory signaling transduction pathways in OSNs, adapted from [3,23], published by Cell Press, 1991 and Annual Reviews, 2002. Adcy, adenylyl cyclase; cAMP, cyclic adenosine monophosphate; CNG, cyclic nucleotide gated channel; Gα_olf_, olfactory G protein alpha subunit.

The basic principles for discriminating more than 10,000 different odorants including odorants released from foods, environmental odorants, and body odorants were not understood until Richard Axel and Linda Buck (Nobel Laureates in Physiology/Medicine, 2004) have jointly clarified how our olfactory system works in their pioneering study. They discovered a large gene family, comprised of some 1000 different mouse genes that give rise to an equivalent number of OR types. These receptors are located on the OSNs, which scatter within the nasal epithelium and recognize the inhaled odorant molecules [3]. ORs are used in a combinatorial manner and therefore are capable of discriminating a myriad of odorants [21,22]. Upon odorant binding, ORs couple to Gα_olf_ and then activate Adcy3. Adcy3 subsequently catalyzes the generation of cAMP from ATP to open cyclic nucleotide-gated (CNG) ion channels. cAMP is the key messenger in the initial phase of odorant detection (Figure 1). Several other intracellular second messengers, such as calcium, cyclic guanosine monophosphate, and inositol-1,4,5-trisphosphate (IP_3_) may also regulate secondary events upon odorant detection [23].

## 3. ORs Are Not Only Expressed in OE

Shortly after the pioneering discovery of the rodent OR gene family in the OE, scientists identified ORs in mammalian germ cells [24,25,26]. In many non-chemosensory tissues and cells, ectopic expression of ORs has currently been discovered. For example, dozens of ORs are ectopically expressed in lung, testis, heart, and liver [27,28]. Using the oligo DNA microarray analysis, Park et al. discovered that as many as 304, 133, and 96 different OR isoforms were differentially expressed in visceral, subcutaneous adipose tissues and skeletal muscle, respectively, during obesity progression [29]. Non-chemosensory tissues may violate the “one neuron-one receptor” rule stating only one OR is expressed in each olfactory neuron cell of the OE since more than one OR are found to be expressed in sperms, myoblasts, and other cell types [9,30]. In addition, OR downstream functional signaling molecules such as Gα_olf_, Adcy3, UDP-glucuronosyltransferases, receptor expression enhancing protein 1, and receptor transporter proteins 1 and 2 are found to be expressed in many non-chemosensory tissues and cells [31,32]. These broad tissue distributions of ORs and their downstream signaling molecules including Gα_olf_, Adcy3, UDP-glucuronosyltransferases, receptor expression enhancing protein 1, and receptor transporter proteins 1 and 2 suggests OR-mediated signaling cascade may serve important roles in many physiological and pathophysiological processes beyond smell [33,34].

## 4. Biological Functions of Ectopic ORs Responding to Odorant Compounds in the Non-Chemosensory Tissues

Over the last three decades, it has become increasingly clear that ORs are not merely pure olfactory receptors, but general chemoreceptors possessing physiologically and pathophysiologically meaningful utilities such as regulation of sperm chemiotaxis [5], energy metabolism [35], chronic skin disease [14], and cancer development [19]. Growing evidence suggests that a diverse array of flavor compounds occurring in foods activate corresponding ORs and therefore regulate distinct physiological processes [12,15,16,36,37,38]. In the following section, we discuss various ectopic ORs with regard to their key physiological function and signaling in response to odorant compounds in several tissues including testis, gut, kidney, skin, lung, heart, skeletal muscle, liver, adipose, pancreatic islets, brain, and cancer tissues.

### 4.1. Role of Ectopic OR in the Testis

From a historical perspective, the testicular ORs were the first ectopic ORs recognized to exert physiological roles. In 1992, Parmentier et al. reported the existence of human ORs in sperm cells [24]. So far, 20~66 ORs are estimated to be expressed in mammalian testis [24,26,39]. In addition, main components of the carnonical olfactory signaling pathway, such as Adcy3 and CNG ion channel, are expressed in sperm and testis [39,40], indicating that the ORs expressed in the testis may utilize the cAMP-Ca^2+^ signaling pathway to regulate testicular function.

**Figure 2:**
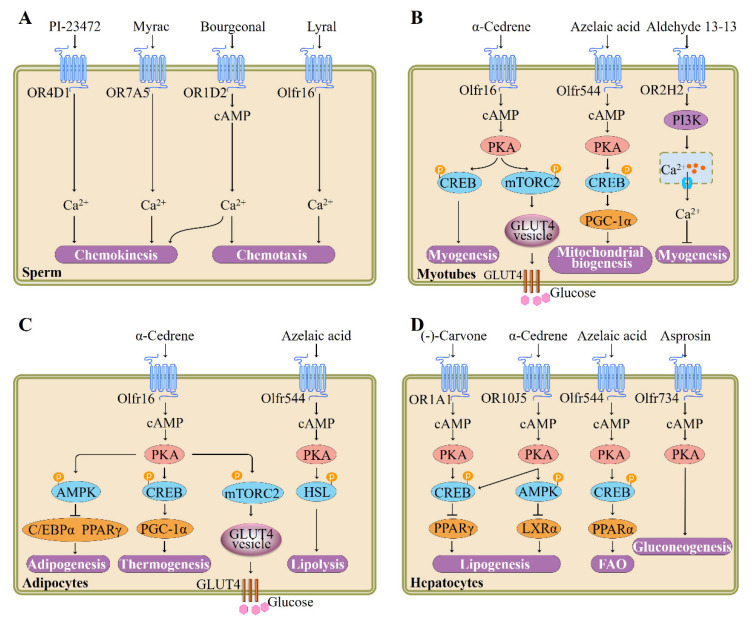
Summary of signaling pathways and functions regulated by ectopic ORs in sperm, myotubes, adipocytes, and hepatocytes. (**A**) Activation of OR4D1, OR7A5, OR1D2, or olfr16 modulates sperm chemotaxis or chemokinesis, adapted from [5,42,43], published by AAAS, 2003; American Society for Biochemistry and Molecular Biology, 2011; The Company of Biologists, 2004. (**B**) Stimulation of olfr16 by α-cedrene in myotubes promotes myogenesis and increases GLUT4-mediated glucose uptake. In addition, olfr544 and OR2H2 activation induces mitochondrial biogenesis and inhibits myogenesis, respectively, adapted from [36,44,45], published by Wiley, 2018; MDPI, 2014 and Elsevier, 2018. (**C**) Regulation of adipogenesis, thermogenesis, GLUT4-mediated glucose uptake, or lipolysis by olfr16 or olfr544, adapted from [18,35], published by MDPI, 2018 and MDPI, 2017. (**D**) ORs (OR1A1, OR10J5, olfr544, or olfr734) can modulate hepatic lipogenesis, fatty acid oxidation (FAO), or gluconeogenesis, adapted from [12,46,47], published by Elsevier, 2015; Nature Portfolio, 2017 and Cell Press, 2019. AMPK, adenosine monophosphate (AMP)-activated protein kinase; cAMP, cyclic adenosine monophosphate; CREB, cAMP-responsive element-binding protein; C/EBPα, CCAAT/enhancer-binding proteins α; HSL, hormone-sensitive lipase; GLUT4, glucose transporter type 4; mTORC2, mammalian target of rapamycin complex 2; PKA, protein kinase A; PPAR, peroxisome proliferator-activated receptor; PI3K, phosphoinositide 3-kinase; PGC-1α, peroxisome proliferator-activated receptor γ coactivator 1-alpha.

Hatt et al. subsequently provided the first evidence that testicular OR participates in sperm motility activation and chemiotaxis. They showed that human sperm exhibits elevated swim speeds and directed movements responding to bourgeonal, a flowery compound which has been found to be the most powerful agonist of OR1D2 (also known as hOR17-4). The cAMP-mediated Ca^2+^ signal may be responsible for this bourgeonal-induced cellular response [5] (Figure 2). Spehr et al. found that in human sperm stimulation of OR4D1 and OR7A5 by odorants PI-23472 and Myrac, respectively, induces characteristic Ca^2+^ responses that correlate with a stimulus-specific motility pattern [41] (Figure 2).

Another kind of testicular OR, the olfr16, is also known to regulate sperm–oocyte chemotaxis. Fukuda et al. confirmed that the olfr16 functions as a chemosensor in mouse sperm and that activation of olfr16 by floral odorant lyral causes an increase in intracellular Ca^2+^ and modulates flagellar configuration, resulting in chemotaxis [42] (Figure 2). In addition to their role in sperm chemotaxis and chemokinesis, testicular ORs responding to specific odorants might also be associated with sperm function, epididymis maturation, and spermatogenesis; nevertheless, further confirmatory studies are needed.

### 4.2. Role of Ectopic ORs in the Muscle

ORs are highly expressed in muscle tissues and cells [9,29,36,45], indicating that ORs may have important physiological roles in these tissues or cell types. Pavlath et al. demonstrated that a wide range of mouse ORs are expressed during myogenesis in vitro and skeletal muscle regeneration in vivo with distinct expression patterns. They specifically investigated the role of olfr16 whose mRNA was upregulated during cell fusion and found that loss of olfr16 suppresses formation of multinucleated myotubes, cell–cell adhesion, and myocyte migration [9]. Olfr16 seems to function importantly in skeletal muscle regeneration and myofiber branching: decreasing olfr16 levels through electroporation of olfr16 siRNA resulted in aberrant regeneration and many branched, unfused myofibers [9]; in contrast, olfr16 overexpression decreases the myofiber branching in dystrophic muscle [9]. Branched myofibers are known to be more susceptible to break and are detrimental for normal muscle physiology [43].

Furthermore, Hatt et al. recently demonstrated the OR2H2 expression in myoblasts and identified aldehyde 13-13 as its ligand (Figure 2). Aldehyde 13-13 was found in human skin secretions, faeces, and saliva [44,45]. It is possible that aldehyde 13-13 is a metabolite or a systemic compound that is absorbed by digestion of food or breathing [45]. Stimulation of OR2H2 by aldehyde 13-13 dose-dependently reduces myoblast fusion. In differentiated human myoblasts, aldehyde 13-13 treatment leads to an increase in Ca^2+^ level. This cellular response is mediated by phosphoinositide 3-kinase (PI3K) signaling cascade, as the PI3K inhibitors LY294002 and wortmannin, not the CNG channel inhibitor or adenylyl cyclase inhibitor, completely abrogate the aldehyde 13-13-elicited Ca^2+^ increase [45].

We recently showed that α-cedrene, which is a natural sesquiterpene component found in cedarwood oils and is used as food additive for flavor adjuvants or enhancement [46], stimulates myogenesis and prevents in vitro myotubes atrophy induced by free fatty acid. These beneficial effects of α-cedrene are mediated by olfr16, as demonstrated by siRNA knockdown experiments. Furthermore, α-cedrene administration decreases muscle wasting in high-fat diet (HFD)-fed mice and increases muscle mass in chow-fed mice. α-Cedrene increases intracellular cAMP levels and protein expression of protein kinase A catalytic subunit (PKA Cα) and Adcy3 induces cAMP-responsive element-binding protein (CREB) phosphorylation, and modulates downstream signaling pathway molecules involved in protein synthesis and degradation such as insulin-like growth factor 1, myostatin, muscle atrophy F-box, and muscle RING finger 1 [36] (Figure 2).

Recently, Lee et al. demonstrated that in cultured skeletal myotubes, stimulation of olfr544 by azelaic acid, a C9 dicarboxylic acid naturally occurring in rye, barley, and other grain foods [47], activates PKA-CREB-peroxisome proliferator-activated receptor γ coactivator 1-alpha (PGC-1α)-extracellular signal-regulated kinase-1/2 (ERK1/2) signaling pathway and stimulates mitochondrial biogenesis and autophagy; these effects of azelaic acid are abolished by olfr544 siRNA transfection (Figure 2). Likewise, administration mice with azelaic acid induces mitochondrial biogenesis and activates the CREB-PGC-1α-ERK1/2 signaling cascade in skeletal muscle tissue of mice; this could not be observed in olfr544^−/−^ mice [20].

Investigation into the function of olfr16 in glucose uptake has been highlighted by recent studies demonstrating that activation of olfr16 by its ligand α-cedrene significantly enhances translocation of glucose transporter type 4 (GLUT4) and glucose uptake in C2C12 cell line and improved HFD-induced glucose intolerance in mice. Molecular analysis revealed that activation of olfr16 increases intracellular cAMP levels, upregulates protein expression of PKA Cα and Adcy3, and induces mammalian target of rapamycin complex 2 (mTORC2) phosphorylation in C2C12 cell line and skeletal muscle tissues of mice [48] (Figure 2).

### 4.3. Role of Ectopic OR in the Adipose Tissue

Obesity and people being overweight are major health hazard for this century, affecting approximately one third of the population in both developing and developed countries. Modification of metabolic efficiency and increasing energy expenditure in adipose tissues represents a crucial strategy to curb obesity [49]. Prior to the functional characterization of ORs in adipose tissues, several lines of indirect evidence supported the notion that ORs have potential physiological roles in the regulation of adipose tissues function. Park et al. performed mode-of-action by network identification analysis and reported that ORs expressed in adipose tissues are possible genetic mediators of HFD-induced obesity progression [29]. Gain-of-function mutation of Adcy3, a downstream signal-transducing molecule in the canonical olfactory signaling machinery, leads to lower body weights and fat mass in mice fed HFD [50]. Similarly, we later demonstrated that haploinsufficiency of Adcy3 leads to obesity in the absence of hyperphagia in mice fed either HFD or chow [51].

The physiological functions of OR in adipose tissues and cells were not explored until relatively recently. To date, the physiological importance of two OR-odorant pairs in adipose tissues or adipocytes have been reported. We recently demonstrated that olfr16, aside from its role in sperms and myotubes [42], appears to regulate energy and lipid metabolism (thermogenesis and adipogenesis) in the 3T3-L1 cell line. Olfr16 activation by sesquiterpene α-cedrene inhibits triglyceride accumulation and increases the oxygen consumption rate, and the effects exerted by α-cedrene are abolished by olfr16 siRNA [9,18]. In agreement with this phenotype, olfr16 activation increases intracellular cAMP levels and protein expression of PKA Cα and Adcy3 and induces phosphorylation of CREB and adenosine monophosphate (AMP)-activated protein kinase (AMPK), along with upregulation of thermogenic genes and downregulation of adipogenic genes [18] (Figure 2). We also showed that long-term α-cedrene administration protects rodents from HFD-induced obesity, and these positive outcomes induced by α-cedrene are largely diminished in Adcy3^+/−^ mice [52]. Furthermore, recent studies demonstrated that in the 3T3-L1 cell line, stimulation of olfr16 significantly increases intracellular cAMP levels, enhances protein expression of Adcy3, PKA Cα, and phosphorylated mTORC2, and therefore promotes GLUT4 translocation and glucose uptake [48].

The physiological roles of ORs in adipose tissues have recently been extended to in vivo metabolism in a knockout mice model. Lee et al. reported that activation of olfr544 by its ligand azelaic acid induces cAMP-PKA-hormone-sensitive lipase (HSL) signaling-mediated lipolysis in adipocytes (Figure 2). Likewise, acute azelaic acid treatment stimulates lipolysis in wildtype mice, but not in olfr544^−/−^ mice. Six weeks of oral administration of azelaic acid attenuates adiposity in HFD-fed wildtype and dramatically induces peroxisome proliferator-activated receptor α (PPAR-α) expression [35].

These studies highlight olfr544 and olfr16 as potential anti-obesity therapeutic targets. Other ORs expressed in adipose tissues, such as olfr1434 and olfr984, are reported to be in association with obesity based on descriptive data; however, functional analysis for these OR genes and identification of their cognate ligands are required [53,54].

### 4.4. Role of Ectopic ORs in the Liver

Liver is known to play a crucial role in the maintenance of systemic glucose and lipid homeostasis. Excess hepatic lipid accumulation causes severe pathophysiological consequences, such as nonalcoholic steatohepatitis and nonalcoholic fatty liver disease. The lipid accumulation in liver largely depends on fatty acid oxidation (FAO) and its synthesis [55]. cAMP-PKA signaling pathways regulate the expression of genes that promote FAO and inhibit lipogenesis. Lee et al. demonstrated that (-)-carvone, a naturally occurring flavor compound which is widely distributed in essential oils of plants such as spearmint [56], reduces the intracellular lipid accumulation of HepG2 cell line and that the effect of (-)-carvone is mitigated in OR1A1-knockdown cells using siRNA. OR1A1 stimulation by (-)-carvone in human hepatocytes increases the intracellular cAMP concentration, but not the Ca^2+^ level, and thus induces PKA activity with subsequent CREB phosphorylation and upregulation of hairy and enhancer of split (HES)-1, a CREB-responsive gene that represses peroxisome proliferator-activated receptor-γ (PPAR-γ) [12] (Figure 1). Furthermore, the group reported that oral administration of (-)-carvone to mice fed HFD for five weeks improves the hepatic steatosis. Activation of olfr43 (mouse homolog of OR1A1) also stimulates the CREB-HES1-PPAR-γ signaling axis in cultured mouse hepatocytes as well as liver of mice [38].

We recently investigated the specific physiological function of OR10J5 in lipid metabolism of human hepatocytes and found that the siRNA-mediated OR10J5 knockdown leads to increased intracellular lipid contents [57]. Treatment of hepatocytes with a natural agonist of OR10J5 α-cedrene significantly reduces the lipid accumulation in human hepatocytes, increases intracellular cAMP levels and protein expression of PKA Cα and Adcy3, and induces phosphorylation of AMPK, CREB, HSL, along with downregulation of lipogenic genes and upregulation of genes associated with FAO in the HepG2 cell line. These beneficial metabolic changes did not occur in OR10J5 knockdown hepatocytes [57] (Figure 2).

In addition to their regulatory roles in hepatic lipid accumulation, OR-mediated signaling pathway regulates hepatic glucose production, a key factor affecting type 2 diabetes development and related metabolic disorders. A recent study by Wang and colleagues reported that olfr734 deficiency significantly decreases the hepatic gluconeogenesis; activation of olfr734 by the identified endogenous ligand Asprosin promote the hepatic glucose production and increases the cAMP level and PKA activity [58] (Figure 2). However, thus far, there are no published studies investigating the role of ORs responding to food-related odorant compounds in hepatic glucose production. As α-cedrene or azelaic acid also significantly improve the glucose tolerance, future study is required to explore the role of ORs responding to α-cedrene or azelaic acid in hepatic gluconeogenesis.

### 4.5. Role of Ectopic ORs in the Gut

**Figure 3:**
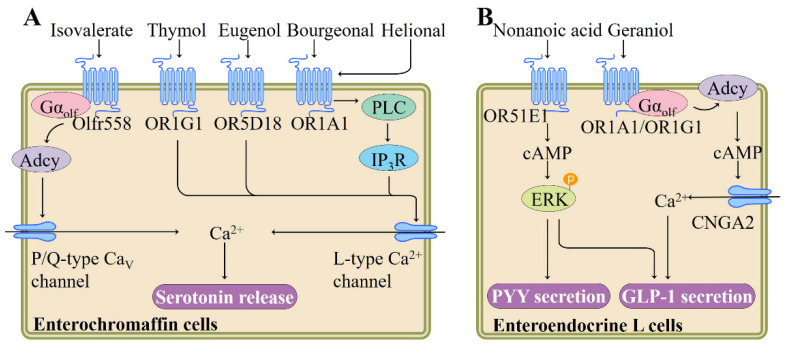
Summary of signaling pathways and functions regulated by ectopic ORs in enteroendocrine cells. (**A**) Activation of olfr558, OR1G1, OR5D18, or OR1A1 enhances the release of serotonin from enterochromaffin cells, adapted from [62,63], published by LIPPINCOTT WILLIAMS & WILKINS, 2013 and Cell Press, 2007. (**B**) Regulation of GLP-1 and PYY secretion from enteroendocrine L cells through OR51E1, OR1A1, or OR1G1, adapted from [64], published by Philadelphia, PA: W.B. Saunders, 2017. Adcy, adenylyl cyclase; CNGA2, cyclic nucleotide gated channel subunit alpha 2; cAMP, cyclic adenosine monophosphate; ERK, extracellular signal-regulated kinase; IP_3_R, inositol-1,4,5-trisphosphate (IP_3_) receptor; Gα_olf_, olfactory G protein alpha subunit; GLP-1, glucagon-like peptide 1; PLC, Gq-phospholipase C; PYY, peptide YY.

In responding to various stimuli, such as dietary nutrients and gut microbial metabolites, diverse enteroendocrine cells within the gut epithelium synthesize and secrete a series of hormones, such as peptide YY (PYY), glucagon-like peptide 1 (GLP-1), and serotonin, and thereby mediate the body’s energy homeostasis [59,60]. Gastrointestinal enterochromaffin (EC) cell is the most abundant endocrine cell type in the body and produces more than 90% of body serotonin by acting as sensors in response to mechanical stimulation and nutrients including fatty acids and glucose [61]. Braun et al. performed reverse transcription-polymerase chain reaction (RT-PCR) and reported the expression of 4 ORs, namely hOR17-210 (OR1E3), OR1G1, hOR17-7/11 (OR1A1), and OR73 (OR5D18) in microdissected human mucosal EC cells and in BON cells, a human EC cell-derived cell line [62]. They found that odorant ligands, such as bourgeonal (floral, lily-of-the- valley), helional (brown algae), eugenol, and thymol, not only increase intracellular Ca^2+^ concentration, but also elicit release of serotonin through exocytosis by activating corresponding ORs expressed in BON cells, although the downstream signaling pathway governed by these ORs remain unclear [62] (Figure 3). Thymol naturally occurs in thyme and is traditionally used as a spice in foods for centuries. Eugenol has been detected in a variety of plant essential oils, especially nutmeg essential oil and clove essential oil [62].

It is well established that gut microbiota plays a predominant role in human health by modulating the metabolism of dietary components such as carbohydrates, protein, and phytochemicals [65]. Recent study has indicated that gut microbiota-derived metabolites could act as signaling molecules through ORs. By using the murine intestinal organoid technology, the role of ORs in EC cells was further investigated by Bellono and colleagues. In an attempt to identify molecules that activate serotonin-producing EC cells, they found that isovalerate, a volatile fatty acid produced by gut microbiota, can induce EC activation. In EC cells, isovalerate triggers a Gα_olf_-Adcy signaling pathway (analogous to the canonical signaling cascade within OE), leading to Ca^2+^ influx via downstream Ca_V_ channels, which is required for serotonin release (Figure 3). Using clustered regularly interspaced short palindromic repeats (CRISPR)/CRISPR-associated protein 9 (Cas9) system to disrupt the olfr558 gene in Chromogranin A-GFP intestinal organoids abolishes isovalerate-evoked responses, implying that in EC cells, olfr558 is necessary for isovalerate signaling [63]. Considering that EC cell-derived serotonin is involved in visceral hypersensitivity disorders, nausea, and gastrointestinal dysmotility [61], these results indicate that manipulation of the release of serotonin via olfr558 may be of therapeutic interest.

Of the intestinal enteroendocrine cells, type L cells also express a range of ORs and secrete diverse gut hormones following odorant-mediated OR activation. For example, treatment of L cells with nonanoic acid, a naturally occurring flavor compound present in a variety of foods including beer, citrus fruits, and honey, dose-dependently stimulates the segregation of anti-diabetic hormones GLP-1 and PYY, along with the cAMP production and phosphorylation of ERK (Figure 3). OR51E1 is required for these nonanoic acid-induced responses in L cells, as evidenced by siRNA knockdown experiments. Nonanoic acid administration significantly increases the circulating GLP-1 levels in normal rats [64]. Similarly, Kim et al. found that geraniol, a monoterpene alcohol component present in lemons, grapes, and geraniums, stimulates GLP-1 release in NCI-H716 cell, an enteroendocrine L cell line, and this cellular response of geraniol is inhibited by the application of Adcy inhibitor SQ22536 or siRNA targeting OR1A1, OR1G1, Gα_olf_, or cyclic nucleotide gated channel subunit alpha 2 (CNGA2) (Figure 3). Moreover, in support of this, oral administration of geraniol to db/db diabetic mice increases plasma GLP-1 level and improves glucose homeostasis [66]. Together, these studies indicate that L cell-expressed ORs play a regulatory role in the maintenance of glucose homeostasis via inducing the secretion of specific gut hormones.

Olfr78 has recently been proposed to be associated with the regulation of intestinal inflammation. Olfr78 and its human ortholog OR51E2 are found to be highly expressed in mouse colon and human intestine, respectively, and the expression of olfr78 is downregulated in mouse colitis models induced by trinitrobenzene sulfonic acid or dextran sodium sulfate. Furthermore, administration of dextran sodium sulfate to olfr78^−/−^ mice increases the mRNA expression of proinflammatory cytokine interleukin-1β [67]. It will be of great interest to delineate the exact role of olfr78/OR51E2 and their corresponding ligands in intestinal inflammation and underlying mechanism in further study.

### 4.6. Role of Ectopic ORs in the Kidney

Pluznick et al. found that six individual ORs and major components of olfaction, including Adcy3 and Gα_olf_, are expressed in the kidney [31]. They further demonstrated that olfr78 is expressed in the afferent arteriole of renal juxtaglomerular apparatus, which is the site of renin (a potent vasoconstrictor) secretion, and identified two short-chain fatty acids (SCFAs) propionate and acetate as ligands for olfr78 [7]. Propionate and acetate are the major endogenous metabolites of dietary carbohydrates by gut microbiota and are also naturally present in various foods. Propionate induces the release of renin in isolated juxtaglomerular apparatus ex vivo, and this effect is abolished by olfr78 deficiency. Supporting this, olfr78^−/−^ mice manifest lowered baseline blood pressure and plasma renin level [7]. The group later reports that another renal OR olfr1393, which is specifically expressed in the proximal tubule of kidney, modulates the renal glucose handling. Olfr1393^−/−^ mice have improved glucose tolerance, normal blood insulin and glucose levels, and mild glycosuria. In concert with these phenomena, the luminal distribution of sodium glucose cotransporter-1, a key renal glucose transporter, is lower in Olfr1393^−/−^ mice compared with that in wildtype mice [68], despite that the precise signaling cascade by which olfr1393 regulates the sodium glucose cotransporter-1 trafficking is not yet understood.

Moreover, OR11H7 and OR51E1 are found to be expressed in the HK-2 human proximal tubule cell line and can be activated by SCFA isovaleric acid. Isovaleric acid is naturally present in foods like apricot, brown algae, beef, and citrus fruits [69]. PCR and Western blot experiments revealed that OR51E1 colocalizes with Gα_olf_ and Adcy3, both of which are canonical olfactory signaling components. Isovaleric acid induced a transient elevation of intracellular Ca^2+^ in HK-2 cells; this isovaleric acid-elicited response was mediated by Adcy3 and extracellular Ca^2+^ [32]. Nevertheless, the physiological significance of OR51E1 and OR11H7 activation responding to isovaleric acid in renal remain unclear and need to be investigated in future experiments.

### 4.7. Role of Ectopic ORs in the Skin

**Figure 4:**
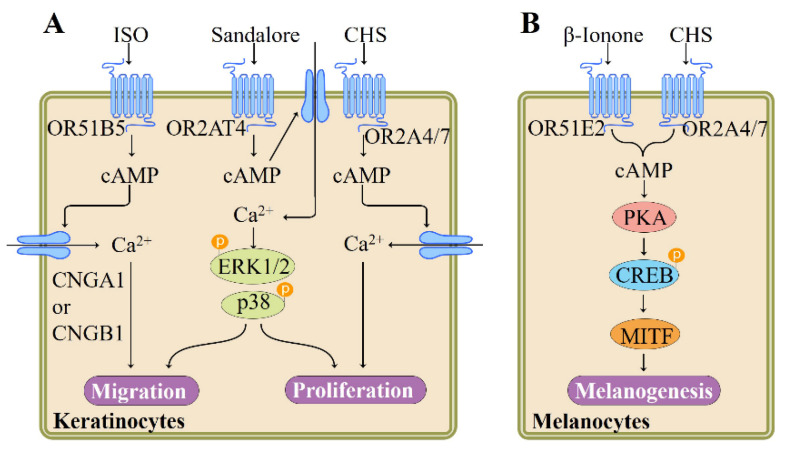
Summary of signaling pathways and functions regulated by ectopic ORs in skin cells, adapted from [74], published by Elsevier, 2018. (**A**) Activation of OR51B5, OR2AT4, or OR2A4/7 increases the migration and proliferation of keratinocytes, adapted from [75,76], published by NATURE PUBLISHING CO., 2019 and Wiley, 2017. (**B**) Both OR51E2 and OR2A4/7 activation enhances melanogenesis, adapted from [77,78], published by Nature, 2018 and American Society for Biochemistry and Molecular Biology, 2016. cAMP, cyclic adenosine monophosphate; CREB, cAMP-responsive element-binding protein; CNGB1, cyclic nucleotide gated channel subunit beta 1; CNGA1, cyclic nucleotide gated channel subunit alpha 1; ERK1/2, extracellular signal-regulated kinase-1/2; MITF, microphthalmia-associated transcription factor; PKA, protein kinase A; p38, p38 mitogen–activated protein kinases.

Skin, the largest organ in the body, not only serves as an effective barrier to protect the body from diverse environmental stimuli including ultraviolet radiation and mechanical stress [70], but also functions as a sensory organ expressing multiple sensory receptors [71]. As the major cell type of the epidermal layer, keratinocytes functionally express a variety of different ORs. For instance, Busse et al. reported that a series of ORs including OR2AT4 are expressed in human keratinocytes. Specific activation of OR2AT4 by Sandalore, a synthetic sandalwood odorant, enhances proliferation, migration, and regeneration of human epidermal keratinocyte in vitro and wound re-epithelialization ex vivo. In cultured human keratinocytes, Sandalore induces Ca^2+^ signal via OR2AT4, as confirmed by siRNA knockdown experiments. The stimulation of OR2AT4 triggers a cAMP-mediated pathway and phosphorylation of p38 mitogen–activated protein kinases (p38 MAPK) and ERK1/2 [72] (Figure 4). Supporting the involvement of OR2AT4 in the skin wound healing process, Kim et al. demonstrated that a 10-lipid mixture representing major lipids of *Chamaecyparis obtuse* plant extract, induces human β-defensin-3 and cathelicidin LL-37, two key players in the wound healing process, in both mouse skin and primary human keratinocytes, and that this effect is mediated through the OR2AT4 as the 10-lipid mixture-induced expression of human β-defensin-3 and LL-37 is inhibited by OR2AT4 siRNA [73].

In addition to keratinocytes, OR2AT4 was also found to be expressed in hair follicle, a specific miniorgan appendage anchored in the subcutis of skin [76]. Hair follicles periodically undergo repetitive cycles of growth (anagen), apoptosis-driven regression (catagen), and relative quiescence (telogen) [79]. Specific stimulation of OR2AT4 by Sandalore promotes human hair growth ex vivo by increasing generation of the anagen-prolonging factor insulin-like growth factor 1 and decreasing apoptosis. In contrast, silencing of OR2AT4 and co-application of the specific OR2AT4 antagonist Phenirat suppress hair growth [76]. These studies support the use of OR2AT4 agonists in the prevention of hair loss.

Tsai et al. validated the expression of two new ORs, namely OR2A4/7 and OR51B5, in keratinocytes by using RT-PCR and immunocytochemistry and identified isononyl alcohol and cyclohexyl salicylate as ligands for these two receptors, respectively. Both odorant compounds may activate Adcy-cAMP-CNG pathway via corresponding OR2A4/7 or OR51B5, leading to Ca^2+^ influx in keratinocytes, as evidenced by receptor knockdown and inhibitor experiments (Figure 4). Nevertheless, functional characterization underlines that two ORs have different functions: while OR2A4/7 regulates keratinocyte proliferation and cytokinesis, OR51B5 influences keratinocyte migration and regeneration [75] (Figure 4).

Keratinocytes are known to be involved in the development of atopic dermatitis, a common skin disease [80]. Interestingly, Tham et al. found that a novel OR, OR10G7, is highly expressed in skin biopsy specimens or primary human keratinocytes from patients with atopic dermatitis. They further investigated the function of OR10G7 in primary human keratinocytes and revealed that activation of OR10G7 by corresponding odorant ligand such as eugenol increases the mRNA expression of interleukin-1β and triggers Adcy-cAMP signaling pathway [14].

A recent study by Duroux et al. demonstrated the expression of OR11H4, OR2AG2, and OR10A6 in human skin and in human primary keratinocytes and the involvement of these skin ORs in skin stress response [81]. Phenylethyl alcohol and a phenylethyl alcohol-rich rose extract function as an agonist of these receptors and protect the skin against the impact of stress in an ex vivo skin stress model induced by epinephrine [82]. Phenethyl alcohol occurs naturally in a variety of plants, such as species of allium plants and anise, and is widely used as fragrance ingredient with a rose-honey-like odor [83]. These findings indicate that activation of ORs may serve as a novel therapeutic target to treat skin stress; nevertheless, future confirmatory studies are needed.

Melanin-producing melanocytes, the key sensory cells providing protective pigmented barrier against ultraviolet irradiation, express ORs and canonical olfactory signaling components Gα_olf_ and Adcy3 [78]. In human epidermal melanocytes, OR51E2 activation by β-ionone significantly induces dendritogenesis and melanogenesis and inhibits melanocyte proliferation. β-Ionone originates from carotenoid and occurs naturally in foods and beverages including wine, orange juice, raspberry, and tomato as a key aroma component [84,85]. OR51E2 signaling mechanism in melanocytes involves cAMP, transient receptor potential melastatin family members, Ca^2+^, and activation of PKA and ERK1/2 and p38 MAPK [77] (Figure 4). Similarly, activation of another melanocyte OR (OR2A4/7) by odorant compound cyclohexyl salicylate is reported to enhance melanin biosynthesis and melanocyte differentiation. These physiological responses appear to be mediated by cAMP-PKA-dependent signaling pathway [78] (Figure 4). Together, these findings suggest that activation of OR expressed in skin melanocytes by odorant compounds may represent a novel strategy that regulates melanogenesis.

### 4.8. Role of Ectopic ORs in the Lung and Bronchi

Airway smooth muscle (ASM) is located in the bronchial tree, where it plays a pivotal role in the modulation of bronchomotor tone, and ASM abnormalities are associated with many airway diseases including asthma [86]. Commonly used anti-obstructive drugs for asthma treatment, such as albuterol and fenoterol (both are β2-adrenergic receptor agonists), work by generating cAMP [87].

Human ASM cells express several isoforms of ORs including OR1D2, OR1J1, OR2A1, OR2AG1, OR6A2, and OR51E2 and their recognized downstream signaling components, such as Gα_olf_, Adcy3, CNGA2, and CNGA4 [88,89,90]. Kalbe et al. demonstrated that ORs expressed in human ASM cells regulate pathophysiological processes: OR2AG1 activation by amyl butyrate inhibits the contraction induced by histamine; in contrast, bourgeonal-induced OR1D2 activation results in an increase in contractility of human ASM cells [88]. Amyl butyrate is a flavor compound occurring in apple, banana, apricot, blue cheese, and other foods and beverages, and is added to products as flavoring agent in the food industry [91]. Although OR2AG1 and OR1D2 induce an opposite cellular response, both receptors seem to utilize cAMP-dependent signaling pathway in human ASM cells [88]. The different downstream signaling molecules such as exchange protein directly activated by cAMP and PKA may be responsible for the different cellular response initiated by OR2AG1 and OR1D2; however, this needs to be confirmed via further investigation. Aisenberg et al. later reported that exposure of human ASM cells to OR51E2 ligand acetate or propionate reduces the cellular proliferation and the rate of cytoskeletal remodeling. This cellular event is mediated by the activation of OR51E2, as evidenced by loss-of-function experiment using CRISPR/Cas9 system [89]. In contrast to the aforementioned OR2AG1 and OR1D2, whose activation induces an increase of cAMP level in ASM cells, OR51E2 activation has a negligible effect on cAMP level [88,89]. Recently, Huang et al. demonstrate that nerol, a monoterpene naturally occurring in plant essential oils, is able to activate the OR2W3 to relax ASM in both tissue and cell models [92]. Collectively, these results demonstrate that activation of ORs expressed in ASM cells may represent an alternate therapeutic strategy to treat airflow obstruction in asthma.

Pulmonary neuroendocrine cells are a rare airway epithelial cell population in the lung and are capable of inducing physiological responses via secreting neurotransmitters, amines, and neuropeptides [93]. Gu et al. reported that human pulmonary neuroendocrine cells express a diverse range of ORs in vivo and in primary cell culture and might act as chemosensory cells in human airways [11]. Li et al. demonstrated that primary pulmonary macrophages express eight ORs and their expression is upregulated by the synergistic action of interferon-γ and lipopolysaccharide. Stimulation of these ORs with the odorant octanal further enhances the production of monocyte chemotactic protein-1 [94]. In addition, genetic study on a four generation Indian family with asthma performed by Chakraborty et al. revealed that OR2AG2 and other ORs may contribute to asthma pathogenesis; nevertheless, further study is required to unravel their specific role in asthma [95].

### 4.9. Role of Ectopic ORs in the Cardiovascular System

ORs and their key downstream signaling elements like Gαolf and Adcy3 are also expressed in the heart [85,86]. In human stem cell-derived cardiomyocytes, OR51E1 ligand nonanoic acid induces a negative chronotropy. This observed physiological effect of nonanoic acid is mediated by OR51E1 as indicated by antagonist and siRNA knock-down experiments [86]. Regarding downstream signaling cascade, OR51E1 stimulation may predominantly involve the G protein activation in stem cell-derived cardiomyocytes.

The human orthologue of olfr16, OR10J5, has been demonstrated to be expressed in the aorta and coronary artery as well as in human umbilical vein endothelial cell (HUVEC). Lyral, a ligand of OR10J5, induces HUVEC migration in vitro and enhances angiogenesis in vivo. In HUVEC, lyral is capable of activating the Ca^2+^-dependent protein kinase B (AKT) signaling pathway, which is mediated by OR10J5 as evidenced by knockdown experiments using siRNA [96]. These findings indicate that some ORs may represent a key molecular and cellular regulator of cardiovascular function.

### 4.10. Role of Ectopic ORs in the Pancreatic Islet

α and β cells, the two best characterized cell types among the endocrine cells found in islets, tightly modulate the glucose homeostasis via regulating the exocytosis of insulin and glucagon, respectively. Impairment of α and β cell function including hypersecretion of glucagon by α-cells and decreased insulin secretion by β-cells is a common manifestation of diabetes [97,98].

**Figure 5:**
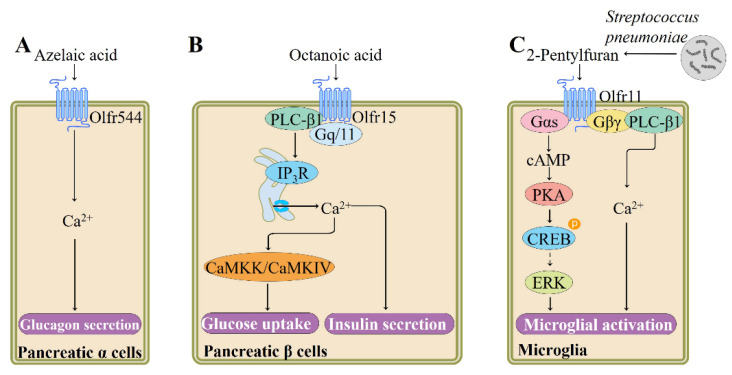
Summary of signaling pathways and functions regulated by ectopic ORs in pancreatic α and β cells and microglia. (**A**) Activation of olfr544 increases the secretion of glucagon from α cells, adapted from [10], published by Elsevier, 2015. (**B**) Stimulation of olfr15 enhances insulin secretion from β cells and glucose uptake, adapted from [99], published by Bentham Science Publishers, 2020. (**C**) Regulation of microglial activation by *Streptococcus pneumoniae*-secreted metabolite acting through olfr110, adapted from [100], published by Elsevier, 2020. cAMP, cyclic adenosine monophosphate; CaMKIV, Ca^2+^/calmodulin-dependent protein kinase IV; CaMKK, Ca^2+^/calmodulin-dependent protein kinase kinase; CREB, cAMP-responsive element-binding protein; ERK, extracellular signal-regulated kinase; IP_3_R, inositol-1,4,5-trisphosphate (IP_3_) receptor; PLC, Gq-phospholipase C.

Glucagon is increasingly appreciated as a potential target to fight type 2 diabetes as it regulates hepatic glucose production [98]. In vitro studies show that activation of OR expressed in α cells promotes secretion of glucagon. For example, Kang and colleagues have shown that olfr544 activation by its ligand azelaic acid in cultured mouse pancreatic α cells (αTC1-9) increases glucagon secretion and intracellular Ca^2+^ mobilization in a concentration- and time-dependent manner (Figure 5). Transfection of pancreatic α cells with olfr544 siRNAs significantly reduces the azelaic acid-induced metabolic changes. As for downstream signaling pathway molecules, expression of Gα_olf_, Adcy3, and olfactory marker protein in pancreatic α cells are confirmed using immunofluorescence staining techniques; however, the precise downstream signaling cascade remains unclear. These results indicate that olfr544 may serve as an important therapeutic target for diabetes [10].

It has been suggested that β cell simultaneously expresses multiple ORs [101,102]. Olfr15 is found to be expressed in a pancreatic β-cell line MIN6 using immunostaining. Activation of olfr15 by its ligand octanoic acid, a medium-chain fatty acid found in beer, whiskey, and various type of cheeses [99], induces glucose-stimulated insulin secretion (GSIS) from MIN6 cells. The octanoic acid-induced enhancement of GSIS is blocked by siRNA-mediated olfr15 downregulation. This olfr15-mediated GSIS enhancement appears to involve Gq-phospholipase C (PLC)-β1-IP_3_-dependent pathway, rather the Gα_olf_-cAMP-PKA pathway, as verified by siRNA and antagonist application experiments [102,103] (Figure 5). Moreover, long-term stimulation of olfr15 with octanoic acid also induces glucokinase expression through the IP_3_-Ca^2+^/calmodulin-dependent protein kinase (CaMKK)/CaMKIV pathway [103].

OR2J3 is found to be expressed in QGP-1 cells, a human pancreatic endocrine cell line, and its specific agonist helional enhances the release of serotonin [104], similar to the effects exhibited by ORs expressed in human EC cells [62]. Nevertheless, it does so using a different mechanism than that used in human EC cells–OR2J3 activation leads to a decreased Ca^2+^ level in QGP-cells, which is mediated by protein kinase G (PKG). Serotonin is known to regulate the release of insulin by serotonylation of signaling enzymes called GTPases in pancreatic β cells [105].

### 4.11. Role of Ectopic ORs in the Brain

ORs are widely distributed in various regions of human and rodent brain, such as selected nuclei of the brainstem, cerebral cortex, and dorsomedial thalamus [106,107]. Altered gene expression of brain ORs is observed in some neurodegenerative diseases, such as sporadic Creutzfeldt-Jakob disease, progressive supranuclear palsy, Alzheimer’s disease, and Parkinson’s disease [108]. These observations suggest that ectopic OR signaling pathway may be associated with the regulation of brain function. Indeed, Koo et al. recently reported that olfr920 is functionally expressed in primary cortical astrocytes and that its activation with short-chain fatty acid isobutyric acid increases intracellular cAMP levels and decreases lipopolysaccharide-induced expression of glial fibrillary acidic protein, suggesting that olfr920 may be a new target to inhibit reactive astrocytosis [109]. Isobutyric acid naturally occurs in various foods, such as apricot, apple, and arctic bramble [91]. In the following study, the group characterized the expression and function of olfr110 in microglia, the important neuroimmune sentinels in the brain. Activation of olfr110 by its ligand 2-pentylfuran, a pathogenic metabolite secreted by *Streptococcus pneumoniae*, regulates reactive oxygen species generation, cytokine production, chemotaxis, and phagocytosis [100]. These microglial activation induced by 2-pentylfuran-olfr110 interaction are mediated through the Gαs–cAMP–PKA–ERK-regulated kinase and Gβγ–PLC–Ca^2+^ pathways, as verified by inhibitor application and receptor knockdown experiments [100] (Figure 5).

### 4.12. Role of Ectopic ORs in the Cancer Tissue

**Figure 6:**
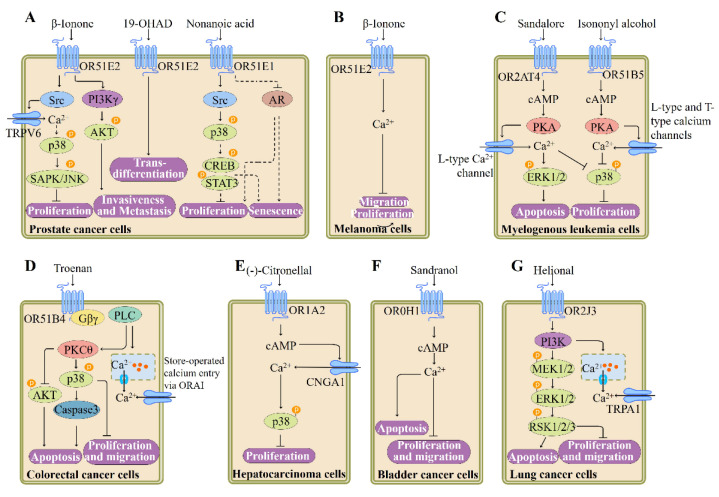
Summary of ectopic OR-induced signaling pathways involved in the regulation of cancer cell function. (**A**) Upon ligand (β-ionone) binding, OR51E2 reduces the proliferation of prostate cancer cells and promotes invasiveness of prostate cancer cells. In addition, activation of OR51E2 enhances cellular transformation, leading to neuroendocrine trans-differentiation. The OR51E1 activation promotes cellular senescence and inhibits the proliferation of prostate cancer cells, adapted from [110,113], published by Elsevier, 2019 and Elsevier, 2018. (**B**) Stimulation of OR51E2 suppresses the migration and proliferation of human melanoma cells [114], published by American Society for Biochemistry and Molecular Biology, 2011. (**C**) Stimulation of OR2AT4 or OR51B5 affects the main physiological processes in human myelogenous leukemia cells, such as proliferation, apoptosis, and differentiation, adapted from [115], published by Wiley, 2017. (**D**) Activation of OR51B4 results in inhibition of proliferation and migration of colorectal cancer cells and induces apoptosis [116], published by Nature, 2016. (**E**) OR1A2 stimulation reduces proliferation of hepatocarcinoma cells, adapted from [117], published by Public Library of Science, 2017. (**F**) OR10H1 is capable of inhibiting bladder cancer cell proliferation and migration and inducing apoptosis when stimulated by its agonist [118], published by Elsevier, 2015. (**G**) Activation of OR2J3 inhibits cell migration and proliferation and induces apoptosis in lung cancer cells [119], published by Frontiers Media S.A., 2018. AKT, protein kinase B; AR, androgen receptor; cAMP, cyclic adenosine monophosphate; CNGA1, cyclic nucleotide gated channel subunit alpha 1; CREB, cAMP-responsive element-binding protein; ERK1/2, extracellular signal-regulated kinase-1/2; MEK, mitogen-activated protein kinase kinase; ORAI, Orai calcium release-activated calcium modulator 1; PKA, protein kinase A; p38, p38 mitogen–activated protein kinases; PI3K, phosphoinositide 3-kinase; RSK, ribosomal S6 kinase; SAPK/JNK, stress-activated protein kinase/c-Jun NH2-terminal kinase; STAT3, signal transducer and activator of transcription 3; Src, sarcoma tyrosine kinase; TRPV6, transient receptor potential vanilloid type 6; TRPA1, transient receptor potential ion channel subfamily A, member 1.

ORs and the downstream targets Adcy3 and Gα_olf_ are identified in cancer tissues and cells, indicating that the OR-mediated signaling pathway plays important roles in these tissues and cell types. Many lines of evidence suggest that OR51E2 could be utilized as a potential target for prostate cancer treatment [19,28,110,111,112,113] (Figure 6). Overexpression of OR51E2 in a mouse model is demonstrated to accelerate the development and progression of prostate cancer along with the loss of PTEN [110]. Upon β-ionone binding, OR51E2 reduces the proliferation of prostate cancer cells by causing phoshorylation of stress-activated protein kinase/c-Jun NH2-terminal kinase (SAPK/JNK) and p38 and promotes invasiveness of prostate cancer cells through Gβγ–PI3Kγ pathway [19,111]. In prostate cancer cells, stimulation of OR51E2 by β-ionone also results in Src kinase-mediated Ca^2+^ influx via transient receptor potential vanilloid type 6 channels [113] (Figure 6). Meanwhile, activation of OR51E2 by newly discovered ligand 19-hydroxyandrostenedione enhances cellular transformation, leading to neuroendocrine trans-differentiation [112]. In the same vein, Kerslake et al. recently demonstrated that OR51E2 is upregulated in prostate adenocarcinoma and by using the GeneMANIA software, they found that this OR interacts with a wide range of genes associated with prostate cancer, such as kallikrein 3, anoctamin 7, arrestin β2, and serine/threonine kinase 3 [28].

Furthermore, the function of OR51E2 is investigated in human melanoma cells: activation of OR51E2 by its ligand β-ionone inhibits the growth and migration of cells derived from vertical-growth phase (VGP) melanoma. β-Ionone treatment leads to an increase in the Ca^2+^ level in cells derived from metastatic melanoma or VGP melanoma, which is mediated by OR51E2 as verified by RNA silencing experiments [114]. This observation suggests that OR51E2 may represent a potential target for the treatment of melanoma (Figure 6).

OR51E1, a paralog of OR51E2, is also demonstrated to be functionally expressed in prostate cancer cells. The OR51E1 agonist nonanoic acid treatment suppresses the proliferation of prostate cancer cells and triggers cellular senescence. Activation of OR51E1 affects the phosphorylation of various protein kinases including p38 and CREB and the expression of androgen-mediated androgen receptor target gene rather than increasing intracellular cAMP or Ca^2+^ levels [120] (Figure 6). Furthermore, another group reported the high expression of OR51E1 in lung carcinoids, indicating that OR51E1 may play a role in somatostatin receptor-negative lung carcinoids [121].

Hatt’s group recently provided the evidence of the functional expression of ORs in several other cancer cell lines such as myelogenous leukemia cells, human hepatocellular carcinoma cell line Huh7, colon cancer cell line HCT116, non-small cell lung cancer A549 cell line, and bladder cancer cell line BFTC905 (Figure 6). Stimulation of OR2AT4 or OR51B5 affects the main physiological processes in human myelogenous leukemia cells, such as proliferation, apoptosis, and differentiation and increases Ca^2+^ level through cAMP-mediated pathway [115,122] (Figure 6). Activation of OR51B4 by synthetic odorant Troenan results in apoptosis of HCT116 cells and inhibition of cell proliferation and migration, which is associated with Gβγ-mediated PLC signaling activation [116] (Figure 6). Monoterpene (-)-citronellal increases Ca^2+^ level in Huh7 cells via activating an OR1A2-cAMP-dependent signaling pathway as verified by siRNA and inhibitor experiments. Meanwhile, (-)-citronellal stimulation reduces cell proliferation and induces the phosphorylation of p38 [117] (Figure 6). In a bladder cancer cell line, OR10H1 is capable of triggering cell cycle arrest and apoptosis and inhibiting cell proliferation and migration when stimulated by its agonist Sandranol. The downstream signaling pathway of OR10H1 involves calcium influx and cAMP increase [118] (Figure 6). Activation of OR2J3 by its agonist helional enhances cytosolic Ca^2+^ level via a PI3K-mediated pathway, inhibits cell proliferation and migration, and induces apoptosis of A549 cells [119] (Figure 6).

Gao et al. reported that in breast cancer specimens, OR2T6 is overexpressed and that it facilitates the proliferation, migration, and invasion, but suppresses the apoptosis of breast cancer cells in vitro. These effects mediated by OR2T6 are associated with MAPK/ERK signaling pathway and epithelial-mesenchymal transition, as confirmed by the specific OR2T6 overexpression or knock-down in breast cancer cell lines as well as microarray and Western blot analysis [123]. In addition, OR7C1 and OR2B6 have been found to function as a potential marker for colon cancer-initiating cells [124] and human breast carcinoma tissues [125], respectively.

Collectively, these selected examples support the fundamental role of OR in the progression of cancers; however, further studies are necessary for the successful development of OR agonist- or antagonist-based therapeutic strategies.

### 4.13. Role of Ectopic ORs in the Coronavirus Disease 2019

Notably, Kerslake et al. recently demonstrated that a series of ORs are co-expressed with key mediators of severe acute respiratory syndrome coronavirus-2 (SARS-CoV-2) infection such as transmembrane protease serine 2, angiotensin-converting enzyme 2, and cathepsin in peripheral tissues including lung, liver, thyroid, bladder, adipose tissue, heart, pancreas, kidney, colon, prostate, and testis [28]. Given the fact that SARS-CoV-2 infection causes partial or total loss of smell [126], it is interesting to explore whether this co-expression could result in additional COVID-19-related consequences in these peripheral tissues in the future study.

## 5. Ligands of Ectopic ORs

Despite their therapeutic potential and physiological importance, most ORs have no known ligands and are referred to as “orphan” receptors. Deorphanization, i.e., unambiguous pairing of receptors and cognate ligands, is thus an important objective in this field. Several modified heterologous cell lines such as Xenopus laevis oocytes [127], the insect Sf9 cell line [128], HEK293 [129], HeLa, and Hana3A [130] and ex vivo dissociated OSNs have been used in OR deorphanization [131]. Moreover, attempts such as co-transfection of cofactors (such as receptor expression enhancing protein 1 and receptor transporter proteins 1) in heterologous cells greatly facilitate the cell surface expression of some ORs and enhance the identification of ligand for ORs [132].

To date, ligand screens performed on human ORs have led to identification of various different OR-odorant pairs (Table 1). Approximately, 86 types of human ORs have been characterized as already deorphanized receptors after identification of their responding ligands, accounting for 20% of the ~400 ORs in humans (Table 1). Exogenous natural or synthetic odorant compounds and endogenous metabolites such as SCFAs have been demonstrated to be ligands for these ORs. Notably, a variety of key food odorants are reported to activate distinct ORs in vitro cell lines [133] (Table 1). Analysis of a limited number of deorphaned ORs reveals that ORs bind odorants combinatorially. That is, some ORs are broadly tended to multiple ligands and a given odorant may activate multiple OR types. In addition, a specific OR can not only bind structurally related odorants but also is recognized by a series of odorants with different chemical structures. Further study is needed to parse the potential physiological roles of identified OR-odorant pairs.

## 6. Conclusions and Future Perspectives

In recent years, a few ORs have been investigated for their ectopic functions. While informative, most of these initial insights have been gleaned from ex vivo whole tissue preparations or in vitro cell lines, leaving fundamental questions unanswered. Further confirmation of the function of these ectopic ORs in vivo is thus desperately required and pivotal. Establishment of whole body or conditional knockout mice for those genes is beneficial to specify the role of ORs in peripheral tissues, and would immensely accelerate research on putative ectopic functions of ORs in diverse tissues or cells. To date, comparatively low number of mouse ORs (olfr544, olfr16, olfr1393, olfr78, and olfr734) have been investigated for their in vivo ectopic functions in a limited number of studies [7,35,43,47,69,169]. With the implementation of latest cutting-edge genome editing technologies such as the CRISPR/Cas9 system to generate whole-body or conditional knockout mice, the speed of illuminating physiological roles of ORs is more than likely to drastically increase over the coming years. Furthermore, it is more challenging to translate the findings in mouse models to humans, given the fact that the mouse orthologue may not always recognize the same odorant ligands with their human counterpart. Notably, the recent developments in the field of single cell-based omics technologies offer new opportunity to enhance our understanding of the functional relevance of extranasal ORs [170].

The lack of ligands for the most ectopically expressed ORs is one major bottleneck and hampers characterization of ORs function in vitro and in vivo. Up to now, ~80% of human ORs remain orphan receptors with no known ligands. Most of the known ligands for ORs are exogenous odorant compounds occurring in foods and plant essential oils [133]. The illumination of ligands of ORs will undoubtedly help to parse the physiological roles and mechanisms of poorly understood ORs. The increasing abundance of structural and molecular GPCR studies in combination with technologies such as virtual ligand screening and a variety of computational approaches may accelerate the discovery of novel ligands of interest, such as food-derived odorant compounds, for orphan ORs.

The OR-mediated downstream signaling cascades responding to odorant compound stimulation in the non-chemosensory tissues are largely unknown. For odorant perception in olfactory neurons, olfactory signal transduction involves classical OR-Gα_olf_-Adcy3-CNG channel and intracellular Ca^2+^-induced action potential. Some groups insist that this OR-Gα_olf_-Adcy3-CNG pathway also occurs in several non-chemosensory cells or tissues, such as myoblast [9], sperm [5], adipocytes [18], and kidney [7]. Nevertheless, studies revealed that the downstream signaling pathways of ectopic ORs responding to odorant stimulation are more complex than previously thought and may be different from those in the olfactory system. For example, in some cell types including gut enterochromaffin cells and pancreatic β-cells, activation of specific OR by its ligand modulates distinct downstream signaling pathway molecules such as Gq, PLC, and IP_3_ receptors [63,103,104]. An increase in knowledge about OR-mediated downstream signaling pathways in response to odorant activation in nonolfactory tissues could immensely contribute to our comprehension about ectopic function of ORs and possibly gives insight to the development of health-promoting ingredients.

Although our understanding of the function, signaling cascade, and pharmacology of ectopically expressed ORs is inadequate and might be the tip of the iceberg, the advances over the last three decades are breathtaking when considered from a historical perspective. The recognition of the potent roles of ectopic ORs in various tissues and cell types, coupled with the fact that these receptors belong to a highly druggable protein family (GPCRs), unequivocally highlight the potential of ectopic ORs responding to flavor compounds, especially food-derived odorants as a promising therapeutic strategy for various diseases [171]. The coming years will show which of the ectopic OR-ligand pairs prove to be relevant pharmacological targets.

## Figures and Tables

**Table 1:** An excerpt of deorphanized human ORs with corresponding odorant compounds, data from [134,135,136,137,138,139,140,141,142,143,144,145,146,147,148,149,150,151,152,153,154,155,156,157,158,159,160,161,162,163,164,165,166,167,168].

OR	Ligands	Refs
OR1A1	Allyl heptanoate	[135]
	Allyl phenyl acetate	[135,136,137]
	trans-Anethole	[138]
	Benzophenone	[135]
	Benzyl acetate	[135]
	Bourgeonal	[63]
	Cosmone	[139]
	Celestolide	[139]
	Citral	[137,140]
	(+)-Carvone	[135,136,137,138]
	(-)-Carvone	[135,138]
	4-Chromanone	[135]
	(S)-(-)-citronellal	[140]
	(S)-(-)-Citronellol	[140]
	(R)-(+)-Citronellol	[140]
	(-)-β-Citronellol	[135]
	(-)-Carveol	[140]
	Dihydrojasmone	[135]
	(+)-Dihydrocarvone	[135]
	4-Decenal	[140]
	Estragole	[138]
	Ethyl cyclohexanecarboxylate	[138]
	Ethyl hexanoate	[138]
	Ethylphenyl acetate	[138]
	Geraniol	[63,135,140]
	Helional	[63,137,140]
	Heptanal	[140]
	Hydroxy-citronellal	[140]
	3-Heptanone	[135]
	(S)-(-)-Limonene	[138]
	(R)-(+)-Limonene	[137,138]
	Musk ambrette	[141]
	Musk xylene	[141]
	Musk tibetene	[141]
	Muscone	[139]
	Musk xylol	[139]
	Muscenone	[139]
	(-)-Menthone	[138]
	(+)-Menthone	[138]
	3-Mercaptohexyl acetate	[138]
	3-Methyl-2,4-nonanedione	[138]
	2-Nonanone	[138]
	Nonanal	[140]
	Nonanethiol	[135]
	(R/S)-γ-Nonalactone	[138]
	Octanal	[140]
	Octanethiol	[135]
	Octanol	[140]
	Quinoline	[137]
	(R/S)-Octen-3-ol	[138]
	2-Octanone	[135]
	3-Octanone	[135]
	2-Pentylpyridine	[138]
	2-Phenylethyl acetate	[138]
	2-Phenylethanethiol	[138]
OR1A2	Citral	[140]
	(-)-Carveol	[140]
	(R)-(+)-Citronellol	[140]
	(S)-(-)-Citronellal	[140]
	4-Decenal	[140]
	Geraniol	[140]
	Helional	[140]
	Heptanal	[140]
	Hydroxy-citronellal	[140]
	Nonanal	[140]
	Octanal	[140]
	Octanol	[140]
OR1C1	Androstenone	[137]
	Coumarin	[30,137]
	Linalool	[136]
	Nonanoic acid	[137]
OR1D2	Anisyl acetate	[142]
	Allyl cyclohexylpropionate	[142]
	Benzyl isobutyrate	[142]
	Benzyl acetone	[142]
	Bourgeonal	[5,30,63]
	Benzyl propionate	[142]
	Benzyl acetate	[142]
	Benzyl butyrate	[142]
	Canthoxal	[5]
	Cyclamal	[5]
	Citral dimethyl acetal	[142]
	Coranol	[142]
	Citronellol	[142]
	Cassione	[142]
	Citral	[142]
	Citronellyl nitrile	[142]
	Cinnamic alcohol	[142]
	Citronellyl oxyacetaldehyde	[142]
	(S)-(-)-Citronellal	[142]
	Cinnamyl nitrile	[142]
	Clonal	[142]
	Dihydromyrcenol	[142]
	Dimethyl ethyl phenyl carbinol	[142]
	Dihydroisojasmonate	[142]
	2,6-dimethyl-7-octen-2-ol	[142]
	(E),(E)-2,4-Decadienal	[142]
	δ-Dodecalactone	[142]
	γ-Dodecalactone	[142]
	9-Decen-1-ol	[142]
	Diethyleneglycol hexyl ether	[142]
	5-Decanol	[142]
	γ-Decalactone	[142]
	δ-2-Decenolactone	[142]
	δ-Decalactone	[142]
	Ethyl heptanone	[142]
	Ethyl cinnamate	[142]
	Ethyl p-anisate	[142]
	Ethyl linalool	[142]
	Ethylene Glycol Monophenoxyacetate	[142]
	Empetal	[142]
	Ethyl phenyl glycidate	[142]
	Florymoss	[142]
	Frutonile	[142]
	Floralozone	[5]
	Geraniol	[142]
	Geranyl propionate	[142]
	Geranyl butyrate	[142]
	Geranyl acetate	[142]
	Hypo-lem	[142]
	Heptanal	[142]
	Hepto	[142]
	Iso jasmone	[142]
	Jasmatone	[142]
	Linalool	[142]
	Lilial	[5]
	Methyl naphthyl ketone	[142]
	Mefrosol	[142]
	Methyl-trans-cinnamate	[142]
	Myrcenol	[142]
	Milk lactone 2067	[142]
	Methyl tuberate	[142]
	7-Methylindole	[142]
	β-Methylphenylethylamine	[142]
	5-Methylindole	[142]
	para-Methoxyacetophenone	[142]
	1-Methylindole	[142]
	Methyl nicotinate	[142]
	3-Methyl-3-nonanol	[142]
	1-Nonanol	[142]
	Nerolidyl acetate	[142]
	Nonalactone	[142]
	cis-6-Nonen-1-ol	[142]
	trans-2-Nonenal	[142]
	Neryl acetate	[142]
	δ-Nonalactone	[142]
	Nonanal	[142]
	Octanal	[142]
	3-Octyl acetate	[142]
	Quintone	[142]
	cis-5-Octen-1-ol	[142]
	7-Octen1-ol	[142]
	1-Octen-3-ol	[142]
	1-Octyl-2-pyrrolidone	[142]
	Petiole	[142]
	Pivarose	[142]
	Phenyl acetaldehyde	[5]
	3-Phenylbutyraldehyde	[5]
	3-Phenylpropionic aldehyde	[5]
	4-Phenylbutyraldehyde	[5]
	Phenyl ethyl isovalerate	[142]
	Phenyl ethyl acetate	[142]
	β-Phenoxy-ethyl-isobutyrate	[142]
	Rosaphen	[142]
	Tetrahydromyrcenol	[142]
	Tetrahydrolinalool	[142]
	(4-Tert-butylphenoxy) acetaldehyde	[5]
	Tetrahydrogeraniol	[142]
	δ-Tridecalactone	[142]
	Tetrahydro citral	[142]
	δ-Tetradecalactone	[142]
	δ-Undecalactone	[142]
	Undecanal (antagonist)	[5]
	γ-Undecalactone	[142]
	Undecene-2-nitrile	[142]
	Violet nitrile	[142]
	Zinarine	[142]
OR1E3	Acetophenone	[63,129]
OR1G1	Acetophenone	[63,143]
	Benzaldehyde	[143]
	Benzothiazol	[143]
	β-Ionone	[63]
	Camphor	[143,144]
	Capric acid	[143]
	Cinnamaldehyde	[145]
	Citral	[143]
	Coumarin	[143]
	(+/-)-Citronellal	[143]
	Decanal	[143]
	9-Decen-1-ol	[144]
	1-Decanol	[143]
	γ-Decalactone	[143]
	1-Dodecanol	[143]
	Ethyl butyrate	[143]
	Ethyl decanoate	[143]
	Ethyl isobutyrate	[143]
	Ethy-2-methyl propanoate	[134,143]
	Ethyl nonanoate	[143]
	Ethyl octanoate	[143]
	Ethyl vanillin	[143]
	Eugenyl acetate	[145]
	2-Ethyl-1-hexanol	[143]
	Floralozone	[145]
	Geraniol	[63,143]
	Guaiacol	[143]
	Hedione	[143]
	Heptanal	[143]
	Hexanal	[143]
	1-Heptanol	[143]
	1-Hexanol	[143]
	3-Hydroxybutan-2-one	[143]
	Isoamyl acetate	[63,129,143]
	2-Isobutyl-3-methoxypyrazine	[143]
	Jasmonyl	[145]
	Lauric aldehyde	[143]
	Limonene	[143]
	Lyral	[143]
	Maltol	[143]
	Maltyl isobutyrate	[145]
	Manzanate	[145]
	Menthol	[143]
	Methyl decanoate	[143]
	Methyl nonanoate	[143]
	Methyl octanoate	[143]
	2-Methyl pyrazine	[143]
	Nonanal	[143]
	Nonanoic acid	[143]
	2-Nonanol	[143]
	2-Nonanone	[143]
	3-Nonanone	[143]
	1-Nonanol	[143,144]
	Octanal	[143]
	Octanol	[143]
	3-Octanol	[143]
	4-Octanol	[143]
	2-Octanol	[143]
	Phenylmethanol	[143]
	Piperonyl acetone	[143]
	Pyrazine	[143]
	Pyridin	[143]
	Quinoline	[143]
	Safrole	[143]
	S-methylthio butanoate	[143]
	Thiazol	[143]
	Thymol	[63,143]
	Trans-anethol	[143]
	Tridecanal	[143]
	2-Undecanone	[146]
	Vanillin	[143]
OR1L3	α-Damascone	[145]
	Vanilin	[145]
OR2A25	Geranyl acetate	[136,137]
	Quinoline	[137]
OR2A4/7	Cyclohexyl salicylate	[76]
OR2AG1	Amylbutyrate	[147]
OR2AG2	Benzyl acetone	[83]
	Citronellol	[83]
	Cis-3-Hexenol	[83]
	α-Cinnamyl alcohol	[83]
	Geraniol	[83]
	Linalool	[83]
	Nerol	[83]
	Phenyl ethyl alcohol	[83]
	Phenyl propyl alcohol	[83]
OR2AT4	Brahmanol	[73]
	Oxyphenylon(Antagonist)	[73]
	Phenirat (Antagonist)	[73]
	Sandalore	[73]
OR2B11	Cinnamaldehyde	[136]
	Coumarin	[136,137]
	(R)-(+)-Limonene	[137]
	Quinoline	[137]
OR2B3	Eugenyl acetate	[145]
	β-Ionone	[145]
	Nerolidol	[145]
OR2C1	Nonanethiol	[135]
	Octanethiol	[135,136]
OR2H1	Methional	[30]
OR2H2	Aldehyde 13-13	[45]
OR2G2	Cinnamaldehyde	[145]
	α-Damascone	[145]
	Maltyl isobutyrate	[145]
	Vanilin	[145]
OR2J2	Citral	[137]
	Coumarin	[30,135]
	Cyclohexanone	[135]
	1-Decanol	[135]
	2,4-DNT	[137]
	Ethyl vanillin	[137]
	Eugenol methyl ether	[137]
	Eugenyl acetate	[137]
	Helional	[137]
	1-Heptanol	[135]
	cis-3-Hexen-1-ol	[136]
	(+)-Menthol	[137]
	Nonanal	[137]
	1-Nonanol	[135]
	1-Octanol	[135,137]
	Octanethiol	[137]
	Quinoline	[137]
OR2J3	Cinnamaldehyde	[136]
	Citral	[137]
	Coumarin	[137]
	2,4-DNT	[137]
	Eugenol methyl ether	[137]
	Geranyl acetate	[148]
	Helional	[137]
	cis-3-Hexen-1-ol	[136,148]
	1-Octanol	[137]
	Musk xylol	[139]
OR2M2	(-)-β-Citronellol	[134,135]
OR2M3	3-Mercapto-2-methylpentan-1-ol	[149]
OR2M4	Cinnamaldehyde	[145]
	Cresyl methyl ether	[145]
	α-Damascone	[145]
	Estragole	[145]
	Fructone	[145]
	Nerolidol	[145]
	Vanilin	[145]
OR2M7	(-)-β-Citronellol	[135]
	Geraniol	[135]
OR2T2	1-Pentanethiol	[150]
	3-Sulphanyl-1-hexanol	[150]
OR2T4	Lilial	[151]
	α-Pinene	[151]
	Undecanal	[151]
OR2T8	1-Pentanethiol	[150]
OR2T10	Cinnamaldehyde	[145]
	α-Damascone	[145]
	Maltyl isobutyrate	[145]
	Terpinyl acetate	[145]
	Vanilin	[145]
OR2T11	Bis (methylthiomethyl) disulfide	[152]
	1-Butanethiol	[152]
	2-Butanethiol	[152]
	Cyclopentanethiol	[152]
	Ethanethiol	[152]
	Methanethiol	[152]
	2-Methyl-1-propanethiol	[152]
	3-Methyl-2-butanethiol	[152]
	2-Methyl-2-propanethiol (t-butyl mercaptan; TBM)	[152]
	1-Propanethiol	[152]
	2-Propanethiol	[152]
	2-Pentanethiol	[152]
	2,3,5-Trithiahexane	[152]
	Thiolane-2-thiol	[152]
OR2T34	Cinnamaldehyde	[145]
	α-Damascone	[145]
	Estragole	[145]
	Floralozone	[145]
	Fructone	[145]
	Jasmonyl	[145]
	Vanilin	[145]
OR2W1	Acetophenone	[135]
	Allyl phenyl acetate	[135,137]
	trans-Anethole	[138]
	Allylphenyl acetate	[138]
	Benzophenone	[135]
	Benzyl acetate	[135]
	Butyl formate	[135]
	Coffee difuran	[137]
	Coumarin	[135,137]
	(-)-carvone	[135]
	(+)-Carvone	[135,137]
	(-)-β-Citronellol	[135]
	4-Chromanone	[135]
	(R)-(-)-Carvone	[138]
	Cinnamyl acetate	[138]
	Decanoic acid	[135]
	Dihydrojasmone	[135]
	(+)-Dihydrocarvone	[135]
	1-Decanol	[135]
	p, α-Dimethylstyrene	[138]
	Eugenol methyl ether	[137]
	Estragole	[138]
	Ethyl cyclohexanecarboxylate	[138]
	Geraniol	[135]
	Helional	[137]
	Heptanal	[135]
	Hexanal	[135]
	Hexyl acetate	[135]
	1-Heptanol	[135]
	1-Hexanol	[135]
	2,3-Hexanedione	[135]
	2-Heptanone	[135]
	2-Hexanone	[135]
	cis-3-Hexen-1-ol	[136]
	3,4-Hexanedione	[135]
	3-Heptanone	[135]
	(E)-2-Heptenal	[138]
	2-Heptanol	[138]
	Hexyl acetate	[138]
	(S)-(-)-Limonene	[138]
	(R)-(+)-Limonene	[137,138]
	2-Methyl-2-propanethiol (t-butyl mercaptan; TBM)	[152]
	Methyl salicylate	[137]
	3-Mercaptohexyl acetate	[138]
	Methyl cinnamate	[138]
	3-Methylbutylacetate	[138]
	4-Methyl acetophenone	[138]
	Nonanethiol	[135]
	Nonanoic acid	[135,137]
	Nonanal	[135,137]
	1-Nonanol	[135]
	2-Nonanone	[135]
	Nonanal	[138]
	2-Nonanone	[138]
	2-Octanone	[135]
	Octanoic acid	[135]
	3-Octanone	[135]
	Octanethiol	[135,137]
	1-Octanol	[135,137]
	Octanal	[138]
	(R/S)-Octen-3-ol	[138]
	(R/S)-γ-Octalactone	[138]
	Prenyl acetate	[135]
	2-Pentylpyridine	[138]
	2-Phenylethyl acetate	[138]
	2-Phenylethanethiol	[138]
OR2W3	Nerol	[30]
OR2Y1	(-)-Carvone	[134]
OR3A1	Aldehyde TPM	[153]
	Bourgeonal (Antagonist)	[153]
	Cyclosal	[153]
	Foliaver	[153]
	Helional	[153,154]
	Heliotropyl acetone	[154]
	Hydrocinnamaldehyde (Antagonist)	[153]
	Lilial	[153]
	Methyl-hydrocinnamaldehyde	[153]
	Methyl-phenyl-pentanal	[153]
	Methylcinnamaldehyde (Antagonist)	[153]
	Trifernal	[153]
OR4D1	PI-23472	[30,42]
OR4D6	β-Ionone	[85]
OR4D9	β-Ionone	[85]
OR4E2	Amyl acetate	[136]
OR4M1	Acetophenone	[155]
OR5A1	β-Ionone	[85]
OR5A2	β-Ionone	[85]
OR5AC2	α-Damascone	[145]
	Eugenyl acetate	[145]
	Fructone	[145]
	Maltyl isobutyrate	[145]
	Manzanate	[145]
	Vanilin	[145]
OR5AN1	Ambretone	[139,141]
	Ambrettolide	[139]
	Cosmone	[139]
	Cyclopentadecanone	[139,141]
	Cyclopentadecanol	[141]
	Civetone	[141]
	Dihydrocivetone	[141]
	(R)-6,6-Difluoromuscone	[141]
	(R)-7,7-Difluoromuscone	[141]
	(R)-8,8-Difluoromuscone	[141]
	(R)-10,10-Difluoromuscone	[141]
	(R)-6,7-Dehydromuscone (E:Z = 10:1)	[141]
	(R,Z)-6,7-Dehydromuscone	[141]
	(R)-9,10-Dehydro-6,6-difluoromuscone (E:Z = 3:2)	[141]
	(R,E)-10,11-Dehydro-7,7-dilfuoromuscone	[141]
	(R,Z)-10,11-Dehydro-7,7-dilfuoromuscone	[141]
	(R)-5,6-Dehydro-8,8-difluoromuscone (E:Z = 10:1)	[141]
	(R,E)-6,7-Dehydro-9,9-difluoromuscone	[141]
	(R,E)-6,7-Dehydro-10,10-difluoromuscone	[141]
	(R,Z)-6,7-Dehydro-10,10-difluoromuscone	[141]
	Exaltolide	[139]
	Ethylenebrassylate	[139]
	Globanone	[139]
	3-Methylcyclotetradecanone	[139]
	Habanolide	[139]
	Isomuscone	[141]
	Muscone	[139,156]
	Musk xylol	[139,141]
	Musk ketone	[139]
	*racemic*-Muscone	[139,141]
	*l*-Muscone	[139]
	*d*-Muscone	[139]
	Musk ketone	[139,141]
	Musk tibetene	[141]
	(R)-Muscone	[141]
	Muscenone	[139]
	ω-Pentadecalactone	[141]
	Thiacyclopentadecane 1-oxide	[141]
OR5B17	Eugenyl acetate	[145]
	Floralozone	[145]
OR5D3P	Raspberry ketone	[157]
OR5D18	2-Acetyl-3,5(6)-dimethylpyrazine (mixture of isomers)	[158]
	2-Acetyl-3-ethylpyrazine	[158]
	2-Acetyl-3-methylpyrazine	[158]
	Benzene	[137]
	Eugenol	[63]
OR5K1	Citral	[137]
	β-Damascone	[137]
	2,3-Diethyl-5-methylpyrazine	[158]
	2,3-Diethylpyrazine	[158]
	2,3-Dimethylpyrazine	[158]
	2,5-Dihydro-2,4,5-trimethylthiazoline	[158]
	2,5-Dimethylpyrazine	[158]
	2,5-Dimethylpyrazine	[158]
	2-Ethyl-3,5(6)-dimethylpyrazine	[158]
	2-Ethyl-3-methoxypyrazine	[158]
	2-Ethyl-3-methylpyrazine	[158]
	2-Ethyl-5(6)-methylpyrazine, mixture of isomers	[158]
	Eugenol	[137]
	Eugenol methyl ether	[137]
	2-Heptanone	[137]
	2-Isobutyl-3-methoxypyrazine	[158]
	2-Isopropyl-3-methoxypyrazine	[158]
	(R)-(+)-Limonene	[137]
	Lyral	[137]
	2-Methoxypyrazine	[158]
	(2R/2S)-4-Methoxy-2,5-dimethylfuran-3(2H)-one, sum of isomers (Methoxyfuraneol)	[158]
	5H-5-Methyl-6,7-dihydrocyclopenta-[b] pyrazine	[158]
	Nonanal	[137]
	Propanal	[137]
	Quinine	[137]
	2,3,5-Trimethylpyrazine	[158]
	2-Vinylpyrazine	[158]
OR5P3	Acetophenone	[135]
	Coumarin	[135,137]
	(-)-Carvone	[135]
	(+)-Carvone	[135,137]
	1-Heptanol	[135]
	1-Hexanol	[135]
	Quinoline	[137]
OR6P1	Anisaldehyde	[136]
OR6X1	(-)-Carvone	[134]
OR7A5	Myrac	[30,42]
OR7C1	Androstadienone	[136]
OR7D4	Androstadienone	[159]
	Androstenone	[159]
OR8B3	(+)-Carvone	[136]
OR8D1	Caramel furanone	[136]
	Sotolone	[30]
OR8H1	Scatole	[160]
OR8K3	β-Damascenone	[137]
	(+)-Menthol	[136,137]
OR10A6	Benzyl acetone	[83]
	α-Cinnamyl alcohol	[83]
	Citronellol	[83]
	Cyclamen aldehyde	[83]
	Cyclemone A	[83]
	Geraniol	[83]
	α-Ionone	[83]
	Nerol	[83]
	Nonadecane	[83]
	Linalool	[83]
	Lyral	[83]
	Phenyl ethyl alcohol	[83]
	Phenyl propyl alcohol	[83]
	3-Phenyl propyl propionate	[83,136,161]
OR10G3	Ethyl vanillin	[136,137]
	Eugenol	[137]
	Vanillin	[136]
OR10G4	Ethyl vanillin	[137]
	Vanillin	[136]
OR10G7	Ethyl vanillin	[137]
	Eugenol	[136,137]
	Eugenyl acetate	[137]
	Eugenol methyl ether	[137]
OR10G9	Ethyl vanillin	[137]
OR10H1	Sandranol	[119]
OR10J1	Dimetol	[30]
OR10J5	Lyral	[135,136,137]
	α-Cedrene	[46]
OR10S1	Lilial	[151]
OR11A1	2-Ethyl fenchol	[136,137]
OR11H4	Isovaleric acid	[162]
	Phenyl ethyl alcohol	[83]
	Phenyl propyl alcohol	[83]
	Skatole	[163]
OR11H6	Isovaleric acid	[162]
OR11H7P	Isovaleric acid	[162]
OR51B4	Troenan	[117]
OR51B5	Isononyl alcohol	[76]
OR51E1	Butyric acid	[137,164]
	Butyl butyryllactate	[135]
	Dodecanoic acid	[164]
	Decanoic acid	[164]
	Dimethyl disulfide	[137]
	2,4-DNT	[137]
	Eugenol methyl ether	[137]
	Eugenyl acetate	[137]
	2-Ethylhexanoic acid (Antagonist)	[164]
	Heptanoic acid	[164]
	Hexanoic acid	[164]
	Isovaleric acid	[136]
	Methyl furfuryl disulfide	[137]
	Methyl salicylate	[137]
	(+)-Menthol	[137]
	3-Methyl-valeric acid	[165]
	4-Methyl-valeric acid	[165]
	4-Methylnonanoic acid	[164]
	Nonanoic acid	[30,135]
	2-Nonenoic acid	[164]
	Octanoic acid	[164]
	1-Pentanol	[137]
	Propanal	[137]
	Pyrazine	[137]
	Pentanoic acid	[164]
	Tetradecanoic acid	[164]
	Trans-2-decenoic acid	[164]
	Tridecanoic acid	[164]
	Undecanoic acid	[164]
OR51E2	Acetate	[7,90]
	AFMK	[113]
	Androstanedione	[113]
	Adenosine-2′,3′-c-phosphate	[113]
	D-Alanyl-d-alanine	[113]
	N-Acetylglutamic acid	[113]
	Bradykinin	[113]
	Epitestosterone	[113]
	Estriol	[113]
	Glycine	[113]
	γ-CEHC	[113]
	L-Glyceric acid	[113]
	Hydroxypyruvic acid	[113]
	L-Histidinol	[113]
	8-Hydroxyguanine	[113]
	β-Ionone	[19,30]
	α-Ionone (Antagonist)	[19]
	α-Ionone (Agonist)	[166]
	Imidazolone	[113]
	Kojibiose	[113]
	2-Ketoglutaric acid	[113]
	19-OH AD	[113]
	Propionic acid	[7,135]
	2-Pyrrolidinone	[113]
	Pelargonidin	[113]
	Palmitic acid	[113]
	Tetrahydrocurcumin	[113]
	Urea	[113]
OR51I2	Isovaleric acid	[167]
OR51L1	Allyl phenyl acetate	[135,136,137]
	Hexanoic acid	[135]
OR52A5	4-Ethyloctanoic acid	[168]
OR52B2	Decanoic acid	[168]
OR52D1	Acetophenone	[143]
	Anisole	[143]
	Benzaldehyde	[143]
	Benzothiazol	[143]
	β-Ionone	[143]
	Butyl butyrate	[143]
	Butyric acid	[143]
	Caproic acid	[143]
	Caprylic acid	[143]
	Cinnamaldehyde	[143]
	Citral	[143]
	Citralva	[143]
	Cyclohexanone	[143]
	Decanal	[143]
	2-Decanone	[143]
	Estragol	[143]
	Ethyl butyrate	[143]
	Ethyl caproate	[143]
	Ethyl heptanoate	[143]
	Helional	[143]
	Heptanoic acid	[143]
	3-Hydroxybutan-2-one	[143]
	Isoamyl acetate	[143]
	Isobutyric acid	[143]
	Isovaleric acid	[143]
	2-Isobutyl-3-methoxypyrazine	[143]
	Lauric aldehyde	[143]
	Methyl heptanoate	[143]
	Methyl octanoate	[30,143]
	6-Methyl-5-hepten-2-one	[143]
	Nonanal	[143]
	Nonanoic acid	[143]
	3-Nonanone	[143]
	2-Nonanol	[143]
	1-Nonanol	[143]
	Octanal	[143]
	3-Octanone	[143]
	Octanic acid	[134,143]
	Octanic acid methyl ester	[134,143]
	Para-anisaldehyde	[143]
	Phenylmethanol	[143]
	Propionic acid	[143]
	Safrole	[143]
	S-methylthio butanoate	[143]
	Thiazol	[143]
	Trans-anethol	[143]
	Trans-cinnamic acid	[143]
OR52E1	Butanoic acid	[168]
OR52E8	3-Hydroxy-3-methylhexanoic acid	[168]
OR52L1	Pentanoic acid	[168]
OR56A1	Decanal	[137]
OR56A4	Decanal	[137]
	Undecanal	[136]
OR56A5	Decanal	[137]
OR56B4	δ-Decalactone	[16]

## Data Availability

Not applicable.
